# Short-term and long-term outcomes of patients with gastric cancer during versus before the COVID-19 pandemic: cohort study using propensity score matching method

**DOI:** 10.1186/s12885-023-11441-w

**Published:** 2023-09-28

**Authors:** Yong Sun, Chao Chen, Lei Hou, Enhong Zhao

**Affiliations:** Department of Gastrointestinal Surgery, Affiliated Hospital of Chengde Medical University, No.36 Nanyingzi Street, Chengde, 067000 Hebei China

**Keywords:** COVID-19 pandemic, Gastric cancer, Short-term outcomes, Long-term outcomes

## Abstract

**Background:**

The negative effects of the novel coronavirus disease 2019 (COVID-19) pandemic on patients with gastric cancer are poorly understood. This study was designed to compare the short-term and long-term outcomes of patients with gastric cancer in the same period before and during the COVID-19 pandemic.

**Methods:**

We retrospectively collected consecutive patients with definite diagnosis of gastric cancer at our center between 1 January and 30 June of 2019 (Before COVID-19) and 2020 (During COVID-19). A comparison was made between the number of patients and their characteristics before and during the COVID-19 epidemic. Propensity score matching (PSM) at 1:1 ratio was performed to evaluate the outcomes of patients that underwent laparoscopic radical gastrectomy in two groups.

**Result:**

The total number of patients diagnosed with gastric cancer during the COVID-19 pandemic increased by 21.4%, compared to that before the COVID-19 pandemic. AII the qualified patients were divided Before COVID-19 Pandemic group (BCP n = 99) and During COVID-19 Pandemic group (DCP n = 118). PSM yielded 81 patients with comparable baseline characteristics into each group. Compared to the BCP group, the DCP group had longer surgery time(P = 0.011), more blood loss(P = 0.015), longer postoperative hospital stay(P = 0.002). No statistical differences were observed in terms of type of resection, number of retrieved lymph nodes (LNs), pathology, short-term and long-term complications (P > 0.05).

**Conclusion:**

Patients diagnosed with gastric cancer during the COVID-19 pandemic had comparable short-term outcomes and long-term complications, but worse peri-operative outcomes, compared to that before the COVID-19 pandemic. Further research is needed to investigate long-term outcomes.

## Introduction

Gastric cancer is the fifth most common cancer and the fourth most common cause of death globally [1]. China has the greatest incidence rate of gastric cancer, with more than 40% of new cases identified worldwide [2]. In Wuhan, China, in December 2019, the novel coronavirus disease 2019 (COVID-19) first emerged. To prevent the viral spread to other Chinese cities and other nations, the Chinese government shuttered Wuhan City on January 23, 2020. Then, a series of measures were taken to contain the epidemic, preventing COVID-19 from spreading across the entire nation. The residents’ daily life and medical conditions have significantly changed since then. Thanks to these timely and effective actions, there has been a significant decline in newly identified and confirmed cases since Feb. 2020.The domestic epidemic was effectively brought under control within almost six months [3].

Measures should be taken to control the number of new COVID-19 cases, whereas these measures can negatively affect the major medical conditions. A cross-sectional study conducted in the United States revealed that weekly diagnoses during the pandemic decreased by 46.4% for gastric cancer [4]. In UK, the quantity of upper endoscopies activities during the pandemic decreased by 12.0% (p < 0.05), and the number of gastric cancers diagnosed weekly decreased by 52% (p < 0.05) [5]. Moreover, a Hongkong study revealed a 51% decrease in endoscopic activity and a 52% decrease in weekly cancer diagnoses for gastric cancer [6]. Furthermore, studies in Japan also showed a significant decrease in the number of gastrectomy and stage I gastric cancer cases, additionally an increase in the number of symptomatic gastric cancer patients [7–9].

Most studies have concentrated on the direct mortality caused by the Covid-19. Specifically, it has been found that patients with cancer are more susceptible to COVID-19 complications and have greater mortality rate [10–13]. However, less focus has been placed on the pandemic’s indirect impact on cancer. The COVID-19 pandemic can lead to delays in diagnosis and treatment of gastric cancer patients, which may negatively affect the prognosis of these patients. Nevertheless, little is known about the adverse impact on the prognosis of patients with gastric cancer, there lack studies that report the clinical outcomes in patients with gastric cancer during the COVID-19 pandemic. Thus, our study intends to evaluate short-term and long-term outcomes of patients by comparing a period of 6 months after the beginning of the COVID-19 outbreak in Wuhan with the same period in the previous year.

## Patients and methods

### Setting

Our institution, Affiliated Hospital of Chengde Medical University, is located in Chengde City, Hebei Province, which is close to Beijing. Our hospital is the center for gastric cancer treatment in the area of Chengde. Although our hospital had adopted a very strict procedure of diagnosis and treatment during the epidemic, there had been no medical disruption. Therefore, the hospital’s medical examinations, operations and chemotherapy went on as usual.

### Patients

**Fig. 1:**
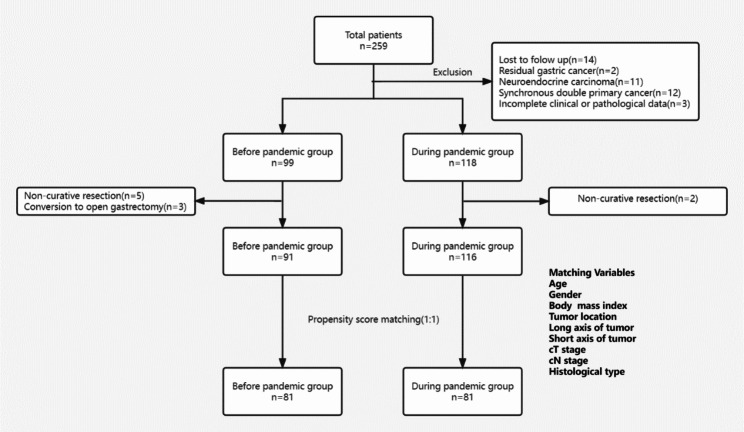
Flowchart showing patient enrollment and PSM matching process

We reviewed 259 patients who were diagnosed with gastric cancer at our hospital between 1 January and 30 June 2019 and 2020.Of these patients, 42 were excluded due to synchronous double primary cancer (n = 12), residual gastric cancer (n = 2), and neuroendocrine carcinoma (n = 11), lost to follow (n = 14), incomplete clinical or pathological data(n = 3). As a result, 217 patients were eligible for inclusion in the present study (Figure 1). The period from 1 to 2019 to 30 June 2019 was classified as before the COVID-19 pandemic, whereas the period from 1 to 2020 to 30 June 2020 was classified as during the COVID-19 pandemic. The demographic and clinicopathological characteristics are comparable between groups and are summarized in Table 1.

To evaluate the short-term and long-term outcomes of patients with gastric cancer within the two periods, another cohort study was performed within these qualified cases. The inclusion criteria were patients who underwent laparoscopic radical surgery for gastric cancer, Patients who underwent open surgery, bypass surgery, resection with D1 dissection, or combined resection of other organs were excluded. All qualified cases were divided into two groups, then matched by Propensity Score Matching at 1:1 ratio to yield comparable baseline characteristics between two groups (Figure 1).

### Data collection and outcome assessment

Demographic characteristics were collected from all the patients, including age, gender, body mass index (BMI), physical status according to the American Society of Anesthesiology (ASA), tumor location and pathological and clinical stage according to the 8th edition of the International Union Against Cancer (UICC) TNM classification [14]. Perioprative outcomes included surgery time, estimated blood loss, number of retrieved LNs, the postoperative hospital stay, postoperative pathological data and outcomes.

Short-term outcomes, which included postoperative complications, reoperation owing to complications, and postoperative mortality, were defined as outcomes within 30 days after surgery. The postoperative complication referred to issues that patients suffered during their hospital stay after surgery, including anastomotic leakage, fever, pulmonary infection, hemorrhage, gastric emptying disorder etc., the Clavien-Dindo classification system was utilized for classifying the postoperative complications system [15].

Long-term outcomes were defined as outcomes obtained at each out-patient visit after discharge, including long-term complications, readmission or reoperation owing to related complications, recurrence, and death during follow-up period.

### PSM

To minimize the impact of potential selective bias, we performed 1:1 matching between the two groups based on the propensity score using a 0.02 caliper width. We used a multivariate logistic regression model to calculate propensity scores for each patient in the two groups. Selected covariates, including age, BMI, gender, ASA score, tumor diameter, tumor location, clinical stage and preoperative histological type. PSM yielded 81 patients with comparable baseline characteristics into each group.

### Statistical analysis

All continuous variables were shown as mean with standard deviation (SD) and were compared by Student’s t test or Mann-Whitney U test. All categorical variables were shown as frequency and percentage and were compared by chi-square test or Fisher’s exact test. When the p-value is less than 0.05, statistical significance was considered. All statistical analyses were performed using SPSS Statistics 25 (IBM, Armonk, NY, USA).

**Table 1:** Clinicopathological characteristics before and during the COVID-19 pandemic

Variables	Categories	Before COVID-19 n = 99		During COVID-19 n = 118		Change(%)	P value		
Age		60.45 ± 8.466		59.75 ± 9.163			0.587		
Gender	Male	75(75.8%)		82(69.5%)		9.3	0.304		
	Female	24(24.2)		36(30.5%)		50			
cT	1	21(21.2%)		26(22.0%)		23.8	0.284		
	2	5(5.1%)		5(4.2%)		0			
	3	23(23.2%)		16(13.6%)		-30.4			
	4	50(50.5%)		71(60.2%)		42			
cN	0	44(44.4%)		40(33.9%)		-9.1	0.106		
	1	15(15.2%)		20(16.9%)		33.3			
	2	23(23.2%)		22(30.6%)		-4.3			
	3	17(17.2%)		36(30.5%)		52.9			
cStage	I	25		30		20.2	0.018*		
	II	23		18		-21.7			
	III	42		68		61.9			
	IV	9		2		-77.8			
cM	0	91(91.9%)		113(95.8%)		24.2	0.241		
	1	8(8.1%)		5(4.2%)		-37.5			
pT	1	21(23.1%)		28(24.1%)		33.3	0.119		
	2	10(11%)		9(7.8%)		10			
	3	30(33%)		25(21.6%)		16.7			
	4	30(33%)		54(46.6%)		80			
pN	0	48(52.7%)		50(43.1%)		4.2	0.138		
	1	14(15.4%)		14(12.1%)		0			
	2	17(18.7%)		22(19.0%)		29.4			
	3	12(13.2%)		30(25.9%)		150			
pStage	I	29(21.9%)		33(28.4%)		12.1	0.036*		
	II	31(34.1%)		25(21.6%)		-19.4			
	III	31(31.4%)		58(50.0%)		87.1			

pT、pN、pStage, patients that underwent radical gastrectomy. *p<0.05.

## Results

### Number and clinicopathological features of patients with gastric cancer diagnosed before and during the COVID-19 pandemic

The total number of patients diagnosed with gastric cancer during the COVID-19 pandemic increased by 21.4% (from 117 to 142), compared to the same period before the COVID-19 pandemic, which was contrary to previous studies [7–9]. Clinicopathological features before and during the COVID-19 pandemic are presented in Table 1. There were significant differences in clinical stage ( cStage p = 0.018), which showed a significant increase in cStage I and cStage III. No significant differences were observed in cT and cN (p = 0.284, and 0.106, respectively). However, the findings revealed a substantial increase in cT4 and cT1. For patients who underwent laparoscopic radical gastrectomy, although no differences were observed in pT and pN (p = 0.119, 0.138, respectively), there were significant differences in pathological stage (pStage P = 0.036). Thus, the results showed a slight increase in pStage I and a significant increase in pStage III, but a reduction in pStage II.

### Baseline characteristic

As shown in Fig. 1, a total of 217 qualified cases underwent laparoscopic radical gastrectomy. Nine cases were excluded due to non-curative resection (7 case), and conversion to open gastrectomy (2 cases). The remaining 207 cases were assigned into BCP group (n = 91) and DCP group (n = 116). Subsequent PSM yielded 81 cases in each group. Table 2 demonstrates that there was no statistical difference of baseline characteristics between BCP group and DCP group.

**Table 2:** Demographics and clinical features between the BCP and DCP groups

Variable		BCP		DCP		P value
		n = 81		n = 81		
Age		60.83 ± 8.077		60.49 ± 9.609		0.801
Gender	Male	59(72.8%)		57(70.4%)		0.175
	Female	22(27.2%)		24(29.6%)		
BMI		21.82 ± 2.78		21.83 ± 2.80		
ASA-PS						1.0
I		1(1.2%)		1(1.2%)		
II		58(71.6%)		58(71.6%)		
III		22(27.2%)		22(27.2%)		
Tumor location						0.859
Upper third		12(14.8%)		13(16.0%)		
Middle third		13(16.0%)		10(12.3%)		
Lower third		56(69.1%)		58(71.6%)		
cT stage						0.545
cT1		20(24.7%)		21(25.9%)		
cT2		4(4.9%)		3(3.70%)		
cT3		19(23.5%)		18(22.2%)		
cT4		38(46.9%)		39(48.1%)		
cN stage						0.545
cN0		37(45.7%)		35(43.2%)		
cN+		44(54.3%)		46(56.8%)		
Clinical stage						0.728
I + II		53(65.4%)		55(67.9%)		
III + IV		28(34.6%)		26(32.1%)		

ASA-PS The American Society of Anesthesiology Physical Status Classification.

TNM staging was based on the recent 8th edition of the AJCC Cancer Staging Manual.

### Short-term outcome

Table 3 exhibits the perioperative outcomes between two groups. There were significant differences in surgery time (p = 0.018), blood loss (p = 0.015), postoperative hospital stay (p = 0.004). No significant difference was observed in type of resection, the number of retrieved lymph node, histological type, pT, pN, and pStage.

**Table 3:** Perioperative outcomes between the BCP and DCP groups

Variable		BCP			DCP			P-value
		n = 81			n = 81			
Surgery time (min)		174.07 ± 28.086			187.65 ± 39.632			0.011
Blood loss(mL)		88.27 ± 62.386			118.27 ± 105.164			0.015
Type of resection								0.496
Proximal gastrectomy		12(14.8%)			13(16.0%)			
Distal gastrectomy		56(69.1%)			58(71.6%)			
Total gastrectomy		13(16.0%)			10(12.3%)			
Histological type								0.292
Well/Moderately		44(54.3%)			38(46.9%)			
Poor/Undifferientiated		37(45.7%)			43(53.1%)			
pT stage								0.596
pT1		20(24.7%)			17(21.0%)			
pT2		7(8.6%)			8(9.9%)			
pT3		27(33.3%)			29(23.5%)			
pT4		27(33.3%)			37(45.7%)			
pN stage								0.162
pN0		41(50.6%)			32(39.5%)			
pN1		14(17.3%)			9(11.1%)			
pN2		15(18.5%)			18(22.2%)			
pN3		71(13.6%)			22(27.2%)			
pStage								0.122
I + II		53(65.4%)			39(48.1%)			
III + IV		28(34.6%)			42(51.9%)			
Number of retrieved LNs		16.68 ± 3.301			18.3 ± 3.750			0.004
Postoperative hospital stay (d)		12.26 ± 2.845			14.36 ± 5.627			0.002

As shown in Table 4, there were 16 cases of complications in the DCP group, including 4 anastomosis leaks, 3 gastric emptying disorder, 5 fever, and 4 pulmonary infections. In the BCP group, there were 11 cases including 1 fever, 3 pulmonary infection, 4 gastric emptying disorder, 2 bowel obstruction, and 1 anastomosis leakage. The incidence of short-term complication was 19.8% and 13.6% in DCP and BCP groups, respectively (P = 0.273). Besides, no significant difference was observed in terms of mild (grades I and II) and severe (grades III and IV) complications between groups.

**Table 4:** Short-term complications between the BCP and DCP groups

				BCP		DCP		P value	
				n = 81		n = 81			
Overall(n, %)^a^				11(13.6%)		16(19.8%)		0.273	
Grade I or II(n, %)^a^				8(9.8%)		12(14.8%)		0.508	
Fever				1(1.2%)		3(3.7%)			
Pulmonary infection				3(3.7%)		4(4.9%)			
Delayed gastric emptying				4(4.9%)		5(6.2%)			
Grade III or IV(n, %)^a^				3(3.7%)		4(4.9%)		>0.999	
Bowel obstruction				1(1.2%)		0(1.2%)			
Anastomosis leakage				2(2.5%)		4(4.9%)			

^a^ Clavien-Dindo’s classification of surgical complication.

### Long-term outcome

In the DCP and BCP groups, the mean duration of follow-up period was 21.25 and 26.29 months, respectively (Table 5). The incidence of long-term complication was comparable between the two groups. There were 15 cases of long-term complications in the DCP group, including 6 abdominal discomfort, 3 anastomosis stricture, 3 bile reflux, and 2 anastomosis inflammation, and 1 gastrointestinal bleeding. In the BCP group, there were 9 cases including 3 abdominal discomfort, 1 gastrointestinal bleeding, 2 bile reflux, and 3 anastomosis inflammation. Additionally, the readmission rate was similar between groups (12.34% vs. 4.94%, p = 0.146). In DCP group, there were 10 readmission cases including 3 anastomosis stricture, 6 abdominal discomfort, and 1 gastrointestinal bleeding. In the BCP group, there were 4 readmission cases due to 1 gastrointestinal bleeding and 3 anastomosis stricture. None of the patients required reoperation during follow-up period. In the DCP group, 4 patients suffered from tumor recurrence, but none of them deceased. In the BCP group, 3 patients survived with tumor recurrence.

**Table 5:** Long-term outcomes between patients in the BCP group and DCP group

	BCP	DCP	P value
	n = 81	n = 81	
Duration of follow up(m)	26.29 ± 14.51	22.25 ± 12.01	0.163
Overall complications	9(11.11%)	15(18.52%)	0.238
Abdominal discomfort	3(3.70%)	6(7.41%)	
Anastomosis stricture	0	3(3.70%)	
Gastrointestinal bleeding	1(1.23%)	1(1.23%)	
Anastomosis inflammation	3(3.70%)	2(2.47%)	
Bile reflux	2(2.47%)	3(3.70%)	
Readmission	4(4.94%)	10(12.34%)	0.146
Reoperation	0	0	
Recurrence	3(3.70%)	4(4.94%)	>0.999
Recease	0	0	

## Discussion

We retrospectively investigated the clinical data of consecutive patients with definite diagnosis of gastric cancer at our center before and during the COVID-19 pandemic. Our study demonstrated that the number of patients diagnosed with gastric cancer during the COVID-19 pandemic increased by 39.8%, compared to before the COVID-19 pandemic, which was inconsistent with previous studies. There was a slight increase in early-stage cancer and a significant increase in advanced stage cancer.

Most studies showed that the number of patients diagnosed with gastric cancer during the epidemic decreased, especially the number of patients with early-stage stomach cancer. Pointed out by Kuzuu et al., the number of patients with newly diagnosed gastric cancer decreased by 26.87%, and the number of cases of stage I gastric cancer decreased 35.51% during the epidemic [4]. Shohei et al. reported the number of patients diagnosed with gastric cancer decreased by approximately 26.2%, and the number of patients with early stage decreased by approximately 38.1% [8]. However, during the epidemic, the number of patients diagnosed with gastric cancer at our center increased by 21.4% on the contrary, among which the number of patients with early-stage gastric cancer increased by 20.2%. The possible reason for that was Chinese government implemented a rigid isolation regime. Before the epidemic, the medical environment in China was very relaxed, and patients had complete freedom to select the hospital of their choice. Some patients prefer to go straight to hospitals in big cities such as Beijing in order to benefit from better medical facilities. During the pandemic, due to strict quarantine policies and regulations, these patients had to be treated at our center. On the other hand, the number of patients with advanced-stage cancers who exhibit symptoms like obstruction increased significantly, which is consistent with previous studies in Japan [7–9].Due to the strict control measures and complicated procedures of hospitals put in place by hospitals during the epidemic, as well as the risk of infection during consultations, patients decided against going to the hospital In order to avoid any unnecessary trouble, even though they have symptoms.

Compared to BCP group, the DCP group had a more blood loss, increased surgery time and longer hospital stay, it did not differ significantly in terms of type of resection, histological type, number of retrieved LNs and pathologic stage. The surgery time in DCP group was comparative longer with a median time of 187.65 min, a more blood loss with a mean of 118.27 mL, and a median hospital stay of 14.36 d. Possible explanations include the fact that patients with gastric cancer during the COVID-19 epidemic had larger tumor lesions, more enlarged lymph nodes, and a more advanced clinical stage, surgeons need to devote more time and greater effort to perform radical resection.

The incidence of perioperative complications is an important factor in evaluating safety of surgery. In this study, no deaths occurred during the perioperative period. The overall complication rates of DCP and BCP in this study were 19.8% and 13.6%, respectively. The morbidity rates, which ranged from 4.8 to 52.6%, were acceptable compared to previous reports [16–19]. Although the rate in DCP group was greater than that in BCP group by 6.2%, the overall complications rate of the two groups did not differ significantly, which may be attributed to the relatively low number of cases. The occurrence rates of anastomotic leak (4.9%vs.2.5%) and gastric emptying disorder (6.2%vs.4.9%) were higher in the DCP group than in the BCP group, with no statistically significant difference as well. However, the postoperative hospital stay was longer in the DCP group than in the BCP group (P < 0.05), which was related to the increased incidence of postoperative complications. Our findings suggested that patients in DCP group had worse short-term outcomes.

The number of harvested LNs is now regarded as an independent prognostic factor in patients with gastric cancer [20–22]. All patients in our study underwent D2 dissection. The median number of LNs was 18.3 in the DCP group, which was a bit higher than that in the BCP group, yet the difference in the number of retrieved LNs was not statistically significant (18.3 vs. 16.7, P>0.05). All patients in two groups underwent R0 resection, and there was no significant difference in number of harvested lymph nodes and pathology. Therefore, patients in the DCP group experienced similar oncological radicalness consequences. On the other hand, the incidence of related complications was comparable between the two groups, and none of patients required reoperation or decease during follow-up period. From an oncology standpoint, patients in two groups had comparable short-term outcomes. The incidence of long-term complication was similar between the two groups. None of the patients required reoperation or deceased during follow-up period. Our findings showed that patients in two groups had comparative long-term complications.

There are limitations of this study that should be noted. Firstly, this is a retrospective single-center study, which might bring selective bias. We performed PSM to minimize the selective bias, but there might still be confounding variables that we missed. Secondly, the sample size might not have been large enough to identify a difference in the complication rate. Lastly, assessing long-term outcomes calls for a longer follow-up period, particularly relapse-free survival and cumulative survival rates.

## Conclusion

Patients diagnosed with gastric cancer during the COVID-19 pandemic had comparable short-term outcomes and long-term complications, but worse peri-operative outcomes, compared to that before the COVID-19 pandemic. Further research is needed to investigate long-term outcomes.

## Data Availability

The datasets analyzed during the current study are available from the corresponding author on reasonable request.

## Abbreviations

COVID-19: Coronavirus disease 2019
LG: Laparoscopic gastrectomy
GC: Gastric cancer
PSM: Propensity score matching
ASA: American Society of Anesthesiology
